# Evaluation of medial olivocochlear efferent system and hearing loss in patients with primary Sjögren’s syndrome

**DOI:** 10.3906/sag-1901-128

**Published:** 2019-12-16

**Authors:** Bülent GÜNDÜZ, Nuriye YILDIRIM, Serdar Can GÜVEN, Emre ORHAN, Recep KARAMERT, Zafer GÜNENDİ

**Affiliations:** 1 Department of Audiology, Faculty of Health Sciences, Gazi University, Ankara Turkey; 2 Division of Rheumatology, Department of Physical and Rehabilitation Medicine, Faculty of Medicine, Gazi University, Ankara Turkey; 3 Department of Otorhinolaryngology, Faculty of Medicine, Gazi University, Ankara Turkey

**Keywords:** Sjögren’s syndrome, hearing loss, medial olivocochlear, efferent system

## Abstract

**Background/aim:**

Autoimmune diseases are a remarkable issue for researchers due to their adverse effects on the auditory system, but for primary Sjögren’s syndrome (pSS) there is little research on the comprehensive audiological findings in literature. The main objective of this study was to investigate the medial olivocochlear efferent functions of subjects with pSS and to examine the audiological findings.

**Materials and methods:**

The study included 36 subjects with pSS and 36 healthy subjects. All the subjects underwent testing including pure tone, speech, and high frequency audiometry; tympanometry and acoustic reflexes; distortion product otoacoustic emissions (DPOAE); and suppression of DPOAE.

**Results:**

The hearing thresholds of the pSS group were higher than those of the control at all frequencies (P < 0.001). Minimal to mild sensorineural hearing loss was observed in 52.77% of all the subjects with pSS. Additionally, all of the subjects had type A curve tympanograms, but the static compliances of the pSS group were lower and the acoustic reflex thresholds were higher than in the control (P < 0.001). In suppression levels of DPOAE, no statistically significant difference was found between the groups (P > 0.05).

**Conclusion:**

The study indicates that because of obtaining normal suppression levels in DPOAE, the medial olivocochlear efferent system is functional in pSS. However, there is a need for more tests, including auditory brainstem response, to evaluate the afferent auditory system in pSS.

## 1. Introduction 

Primary Sjögren’s syndrome (pSS) is an autoimmune disorder characterized by eye dryness and salivary hypofunction due to inflammation of lacrimal and salivary glands [1]. The incidence of pSS in women is higher than in men with the highest incidence of disease at about 40–50 years in women [2–4]. 

Different anatomical regions of the auditory system may be affected by pSS, resulting in symptoms such as otalgia, tinnitus, vertigo, and hearing loss. There is little research on the pathophysiological impact that pSS may have on the human auditory system. While there are no robust findings about the effects of pSS, especially for the outer ear and middle ear, the possible reasons for auditory symptoms are thought to be dryness of the ear canal epidermis, middle and inner ear fluids, and dysfunction of the Eustachian tube [5,6]. On the other hand, there are many hypotheses about the pathophysiological impact of pSS on the inner ear. The inner ear is defined as a sensory organ that contains perilymph and endolymph fluid and it transduces sound waves into senses of hearing to be used by the auditory neural parts. These neurosensory functions are tightly bound to the regulation of molecules in the perilymph and endolymph fluid volume in the inner ear [6]. Change in the volume of the inner ear fluids can disrupt the molecular structure of the fluids and cause hearing loss. Additionally, ototoxic drugs used during the treatment of autoimmune inner ear diseases can cause the damage of intracellular fluids and change the molecular concentrations for physiological mechanisms. Furthermore, autoantibodies are produced in perilymph and endolymph fluids in normal hearing physiology, but in diseases such as pSS they are produced inadequately [5,6]. Some studies showed that the incidence of mild to severe hearing loss in patients with pSS was predicted as 78.38%, though many patients were unaware of their hearing loss [7]. Other studies asserted that patients with pSS have higher hearing thresholds than healthy subjects in the 500–3000 Hz frequency range [8], and recent studies have concentrated on high-frequency hearing loss in pSS [8,9]. While the present paper concentrates on medial olivocochlear system functions in pSS, hearing loss, including high-frequency hearing loss, is roughly evaluated as well.

In the diagnosis of hearing loss and medial olivocochlear efferent system functions, otoacoustic emissions (OAEs) testing is one of the crucial clinical and diagnostic tools, because it reveals the cochlear component of hearing impairment. OAEs are generated by active micromechanics of the outer hair cells (OHCs) in the organ of Corti and they are low-intensity sounds produced by the cochlea as a part of normal auditory processes [10]. Distortion product otoacoustic emissions (DPOAEs) precisely detect cochlear dysfunction as frequency-specific and they are obtained in spite of hearing loss to some degree, so they are preferred for assessment of cochlear activity and medial olivocochlear efferent system functions in the present research.

Moreover, the suppression of OAEs means a fair amount of decrease in the response amplitude with contralateral ear stimulation, which reduces the electromotility of the OHCs and in particular suppresses OAEs. The suppression is related to the efferent auditory pathway, which originates from the superior olivary complex. Since the contralateral sound-induced suppressive effect is mediated by the medial superior olivary complex (MSOC) neurons, contralateral suppression of OAEs gives direct information on MSOC efferent activation. If the contralateral ear is stimulated when noise is present, it can give an idea. This effect is explained by the change of cochlear micromechanics of the MSOC, which can be activated by contralateral acoustic stimulation. The present study therefore explored the suppression of emissions to investigate the medial olivocochlear (MOC) system in pSS. Since the MOC efferent system provides better hearing performance and speech discrimination in noisy environments, disruption of the MOC system, which can be evaluated by suppression of OAEs, causes abnormalities of temporal functions like discrimination, understanding, and lateralization of sounds in a noisy environment [11].

However, there is little research on medial olivocochlear efferent system function in pSS [8,12]. Therefore, this study is the first to evaluate the functions of the MOC efferent system related to neurosensory functions in pSS by using contralateral suppression of DPOAEs. We expected that MOC dysfunction in pSS patients adversely affects neurosensory functions in the inner ear fluids and upper auditory pathway. To reveal the importance of routine audiological evaluations in pSS, the study also investigated other audiological findings extensively. 

## 2. Materials and methods

All the subjects agreed to participate in this randomized controlled study and provided signed informed consent prior to the investigation. In addition to this, the study was performed with institutional decision number 396 for ethical approval. Patients meeting the classification criteria for pSS were evaluated for eligibility [13]. After physical and laboratory examinations, disease activity was measured with the EULAR Sjögren’s Syndrome Disease Activity Index (ESSDAI) and patients with ESSDAI scores of <14 were enrolled. 

The exclusion criteria included high disease activity (ESSDAI ≥14), exposure to noise, genetic hearing loss, neurological disease, tinnitus, vertigo, ototoxic medication, trauma, ear pathologies or ear surgery history, and age over 75 years. A total of 36 pSS patients were enrolled as the study group and 36 age- and sex-matched healthy subjects with normal hearing thresholds were enrolled as the control group. Since hydroxychloroquine (HCQ) is frequently used in pSS and antimalarial drugs are suspected to cause ototoxicity, hearing performance of HCQ users and nonusers were also compared as subgroup analysis in order to investigate possible ototoxic effects of HCQ.

### 2.1. Pure tone-speech audiometry

All the audiological evaluations of the subjects were performed by the same audiologist in a silent cabinet with a GSI-AudioStar Pro clinical audiometer with air conduction thresholds at frequencies of 125–8000 Hz and bone conduction thresholds of 500–4000 Hz. The high-frequency hearing thresholds were found at frequencies of 9000, 10,000, 11,200, and 12,500 Hz. Hearing loss was classified as low frequency (125, 250, 500 Hz), middle frequency (500, 1000, 2000 Hz), high frequency (4000, 6000, 8000 Hz), and very high frequency (9000, 10,000, 11,200, 12,500 Hz). Subjects with a limit of perception below 20 dB HL at all frequencies were defined as normal [14]. The pure tone threshold averages were calculated at 500, 1000, 2000, and 4000 Hz and hearing loss degrees were defined according to established criteria [14]. The speech audiometer was used to evaluate speech recognition thresholds (SRT) and speech discrimination (SD) scores and they were compared between the pSS and control groups. Subjects who had speech discrimination scores above 88% were defined as normal [14].

### 2.2. Acoustic immittance 

Acoustic immittance involves the middle ear pressure, the static compliances, and the presence of ipsilateral and contralateral acoustic reflexes. A 226-Hz tympanometry measurement and ipsilateral-contralateral acoustic reflexes were assessed with the GSI TYPmpStar. 

Subjects with middle ear pressures of 0–100 daPa and compliance values of 0.33 mmHo to 1.33 mmHo were defined as normal. Acoustic reflexes of 75–95 dB at frequencies of 500, 1000, 2000, and 4000 Hz were considered normal [14].

### 2.3. Otoacoustic emissions

DPOAE measurement was performed with an Interacoustic Eclipse 15 using insert earphones. The frequency ratio of the two primary tones (f2/f1) was fixed at 1.22. Stimulus levels were kept at 65 dB SPL for f1 and 55 dB SPL for f2 frequencies. DPOAE measurement at 2f1 – f2 was considered significantly different from the background noise if it exceeded it by at least 3 dB. DPOAEs were obtained between 1 kHz and 6 kHz, and signal-to-noise ratios of these responses were compared between the pSS and control groups. Additionally, white noise at 50 dB SPL was used to stimulate the contralateral ear side during otoacoustic emission measurement to evaluate MOC activities [15–17].

### 2.4. Statistical analyses

SPSS 25 for Windows 7 was used for statistical analyses (IBM Corp., Armonk, NY, USA) and P < 0.001 was considered statistically significant. The variables were investigated using visual (histogram and probability plots) and analytical methods (Kolmogorov–Smirnov/Shapiro–Wilk test) to determine whether or not they were normally distributed. Descriptive analyses were presented using mean and standard deviation for normally distributed variables, while median and interquartile range (25th-75th IQR) were used for abnormally distributed ones. The P-values result from the independent samples t-test in the group that demonstrated normal distribution and the Mann–Whitney U-test was applied for nonparametric situations.

## 3. Results

### 3.1. Clinical findings

After consulting the inclusion and exclusion criteria, we assessed 36 female subjects (72 ears) with pSS and compared them with 36 healthy female subjects (72 ears). The mean age of pSS patients and controls were 51.42 ± 10.21 years (range: 38–64 years) and 49.72 ± 4.19 years (41–59 years) respectively. The subjects in the pSS group were diagnosed about 20 months ago. The clinical profile is summarized in Table 1. These findings are given in the study only as descriptive statistics, so no correlation between the clinical profile and auditory performance of pSS patients was analyzed. Many of the subjects with pSS complained about having communication problems sometimes, including speech discrimination and perception in noisy environments. 

**Table 1:** Clinical features of subjects with pSS (n = 36).

Age at inclusion, years§	51.42 ± 10.21
Duration, months¶	31.0 (5.0–36.0)
Disease characteristics, n (%)	
Arthralgia	35 (97.1)
Fatigue	34 (94.4)
Sicca symptoms	32 (88.9)
Arthritis	8 (22.2)
Systemic involvement	9 (25)
Anti-SSA positivity	20 (55.6)
Anti-SSB positivity	4 (8.3)
ANA positivity	31 (86.1)
RF positivity	5 (13.9)
Biopsy procedure	22 (61.1)
Positive biopsy	22 (100)
Hypocomplementemia	5 (13.9)
Hypergammaglobulinemia	4 (11.1)
ESR, mm/h¶	13.5 (1.0–65.0)
CRP, mg/L¶	4.0 (1.0–23.0)

§ Data presented as mean ± SD; ¶ data presented as median (min–max); ANA, antinuclear antibody; RF, rheumatoid factor; ESR, erythrocyte sedimentation rate; CRP, C-reactive protein.

### 3.2. Audiological findings

The hearing thresholds were analyzed for right and left ears in four groups of frequency ranges: 125–250–500 Hz are low, 500–1000–2000 Hz are middle, 4000–6000–8000 Hz are high, and 9000–10,000–11,200–12,500 Hz are very high frequencies. The hearing threshold averages of pSS patients were compared with the controls for all frequency groups (Table 2).

**Table 2:** Pure tone audiometry results of subjects with pSS and controls

Frequency range	Ear side	pSS,mean ± SD	Controls,mean ± SD	P
Low-frequency average(dB HL) (125, 250, 500 Hz)	Right	9.39 ± 6.29	4.39 ± 3.46	<0.001*
	Left	10.41 ± 4.54	4.30 ± 3.71	<0.001*
Middle-frequency average(dB HL) (500, 1000, 2000 Hz)	Right	14.30 ± 7.18	6.11 ± 4.17	<0.001*
	Left	15.64 ± 5.72	5.41 ± 3.21	<0.001*
High-frequency average(dB HL) (4000, 6000, 8000 Hz)	Right	21.52 ± 9.58	5.78 ± 3.49	<0.001*
	Left	23.05 ± 8.90	6.48 ± 3.43	<0.001*
Very high-frequency average(dB HL) (9000, 10,000, 11,200, 12,500 Hz)	Right	45.62 ± 17.60	22.15 ± 7.49	<0.001*
	Left	44.20 ± 17.26	22.95 ± 9.03	<0.001*

*P < 0.05 (independent samples t-test).

In all the frequency ranges, a statistically significant (P < 0.001) difference was found. Accordingly, the hearing thresholds were obtained from both of the groups, increasing gradually towards the very high frequency range. The most significant difference was calculated for the right ear in very high frequency range between the pSS and control groups, because the thresholds were observed at 45.62 dB in pSS patients while the controls had a threshold at 22.15 dB (P < 0.001). Moreover, minimal to mild sensorineural hearing loss was observed in 52.77% of pSS patients (n = 19) according to pure tone averages. According to a study on very high frequency hearing thresholds in normal-hearing adults (9000–12,500 Hz), 30 subjects (83.33%) had high frequency hearing loss in the current study [18]. 

The speech audiometry results showed a correlation between pure tone averages and SRTs for the two groups. In addition, SRTs were about 5–10 dB higher than pure tone averages. The SD scores were determined with mean and standard deviation of 94.11 **± **5.77% in pSS patients and similarly 96.22 **± **3.57% in the controls. Consequently, the speech audiometry results were correlated with the hearing thresholds for all subjects. 

The tympanometry results of pSS patients and those of the control group were analyzed by middle ear pressure and static compliance values as shown in Table 3. All subjects had type A curves; thus, the middle ear pressures and the static compliances were in the normal range. However, one point to be noted is that the static compliances of the pSS group were lower than those of the control group, and when these static compliances of pSS patients were compared with the controls, a statistically significant difference (P = 0.000) was found between the groups. Likewise, static compliances for the right ears of pSS patients were about 0.49 mmHo, while they were 0.65 mmHo in the controls. On the other hand, no statistically significant difference was found in the middle ear pressures between the groups (P = 0.299, P = 0.804).

**Table 3:** Tympanometry results of subjects with pSS and controls.

	Ear side	pSS, mean ± SD	Controls, mean ± SD	P
Middle ear pressure (daPa)	Right	–8.94 ± 24.19	–3.80 ± 16.87	0.299
	Left	–6.19 ± 23.77	–4.97 ± 17.24	0.804
Static compliance (mmHo)	Right	0.49 ± 0.16	0.65 ± 0.12	<0.001*
	Left	0.51 ± 0.17	0.66 ± 0.13	<0.001*

*P < 0.05 (independent samples t-test).

The acoustic reflex thresholds of the pSS group were higher than those of the control subjects as ipsilateral or contralateral measurements and, related to this, statistically significant differences were found at all frequencies (P = 0.000) (Table 4). Furthermore, in general, the obtained contralateral acoustic reflex thresholds were higher than the ipsilateral ones.

**Table 4:** Acoustic reflex thresholds of subjects with pSS and controls.

Acoustic reflexes thresholds	Ear side	pSS (dB),mean ± SD	Controls (dB),mean ± SD	P
Ipsilateral reflexes				
500 Hz	Right	85.69 ± 3.99	81.94 ± 2.47	0.017*
	Left	86.66 ± 4.78	84.44 ± 2.61	0.042*
1000 Hz	Right	85.97 ± 3.54	81.80 ± 2.43	<0.001*
	Left	86.80 ± 4.16	83.61 ± 2.56	<0.001*
2000 Hz	Right	87.63 ± 3.27	83.05 ± 2.47	<0.001*
	Left	88.19 ± 3.19	83.75 ± 3.01	<0.001*
4000 Hz	Right	90.13 ± 2.23	85.13 ± 1.88	<0.001*
	Left	90.69 ± 2.71	84.58 ± 3.01	<0.001*
Contralateral reflexes				
500 Hz	Right	90.13 ± 3.87	85.27 ± 2.91	0.021*
	Left	91.94 ± 4.18	86.94 ± 3.22	0.011*
1000 Hz	Right	88.88 ± 2.42	83.88 ± 2.42	< 0.001*
	Left	89.30 ± 3.41	84.44 ± 2.87	< 0.001*
2000 Hz	Right	89.44 ± 2.87	84.16 ± 2.80	<0.001*
	Left	89.58 ± 4.03	84.86 ± 3.48	<0.001*
4000 Hz	Right	90.41 ± 3.24	84.58 ± 4.03	<0.001*
	Left	91.66 ± 3.77	85.83 ± 3.48	<0.001*

*P < 0.05 (independent samples t-test).

DPOAE responses were obtained from all the subjects, but there was no significant difference (P > 0.001) (Table 5). Response amplitudes of 1 kHz and 2 kHz were found to be higher than the other frequencies for both groups and ear sides in general. 

For suppression of DPOAEs, if the criterion of an amplitude decrease of >1 dB was taken into account, a suppressive effect was obvious in the pSS and control groups [19]. The contralateral sound caused suppression of the otoacoustic emission responses in subjects with pSS; therefore, normal sensitivity to noise as the quality of sound was perceived. The highest suppression levels were observed at 1 kHz as 2.64 dB for pSS patients and as 2.60 dB for the controls (Table 6); however, there was no statistically significant difference calculated between the groups (P = 0.920). There was also no significant difference at 1 kHz for suppression levels, nor was any significant difference found at the other frequencies as shown by the P-values.

**Table 5:** DPOAE amplitude of subjects with pSS and controls.

	Ear side	pSS (SNR dB)	Controls (SNR dB)	P
1 kHz, mean ± SD	Right	14.58 ± 4.09	13.20 ± 2.58	0.131
	Left	13.86 ± 5.30	14.81 ± 2.66	0.362
2 kHz, mean ± SD	Right	14.76 ± 3.40	11.73 ± 3.04	0.099
	Left	13.21 ± 3.12	12.57 ± 2.59	0.344
4 kHz, mean ± SD	Right	12.53 ± 2.92	9.39 ± 2.41	0.018*
	Left	11.07 ± 3.43	9.72 ± 2.12	0.032*
6 kHz, median (IQR)	Right	7.25 (5.40–7.90)	3.70 (3.10–4.10)	0.068
	Left	5.95 (5.20–7.50)	4.50 (4.10–5.80)	0.016**

*P < 0.05 (independent samples t-test), **P < 0.05 (Mann–Whitney U-test).

**Table 6:** DPOAE suppression level of subjects with pSS and controls.

	Ear side	pSS(dB)	Controls(dB)	P
1 kHz, mean ± SD	Right	2.64 ± 2.12	2.60 ± 1.84	0.920
	Left	2.08 ± 2.42	2.88 ± 2.05	0.134
2 kHz, mean ± SD	Right	1.96 ± 1.90	1.97 ± 2.04	0.980
	Left	2.08 ± 2.00	1.98 ± 1.91	0.817
4 kHz, mean ± SD	Right	2.06 ± 2.01	2.06 ± 2.21	0.955
	Left	2.28 ± 1.58	1.40 ± 2.23	0.191
6 kHz, median (IQR)	Right	0.75 (0.00 to 1.70)	1.00 (0.60 to 2.30)	0.684
	Left	0.55 (–0.70 to 1.50)	1.70 (1.40 to 2.40)	0.122

*P < 0.05 (independent samples t-test), **P < 0.05 (Mann–Whitney U-test).

A total of 13 pSS patients were HCQ nonusers for various reasons (coexisting retinal disease, allergy, not preferred by physician, etc.). Subgroup analyses revealed no significant difference in hearing performance between HCQ users and nonusers (mean hearing thresholds, P = 0.612; mean middle ear pressure, P = 0.869; mean acoustic reflexes thresholds, P = 0.315; mean DPOAE amplitude, P = 0,281; mean DPOAE suppression level, P = 0.685; independent samples t-test). When compared to healthy subjects, HCQ nonusers had impairment similar to the general pSS population in the study (mean hearing thresholds, P = 0.03; mean middle ear pressure, P = 0.128; mean acoustic reflexes thresholds, P = 0.018; mean DPOAE amplitude, P = 0,035; mean DPOAE suppression level, P = 0.452; independent samples t-test).

## 4. Discussion

This study is the first to reveal the functions of the MOC efferent system related to auditory neurosensory functions in pSS and also examine the audiological findings extensively in pSS. 

We found minimal to mild sensorineural hearing loss in more than half of our pSS subjects, possibly resulting from degeneration of inner ear fluids with pSS. Because the cochlea is a sensory organ that transforms auditory inputs to neurosensory functions, this mechanism is tightly dependent on the fluids in the inner ear, which contain several proteins and other molecules. Similarly, the molecules of the inner ear fluids degenerate from secondary Sjögren’s syndrome or Meniere’s disease and thus hearing loss can be observed in subjects with pSS [5]. Accordingly, this study implies that subjects with pSS are more likely to suffer hearing loss than their controls compatible, with the literature.

The main aim of this study was not to evaluate high-frequency hearing loss and the present study included a small number of subjects with pSS according to some other studies in the literature for investigating only high-frequency hearing loss. However, we also examined these findings because of some communication problems among our subjects with pSS. Consequently, most of the subjects with pSS had hearing loss in both ears at very high frequencies (9000–12,500 Hz), which is in accordance with other studies [8,9]. A significant difference at 125–250–500 Hz between the groups was obtained, differently from some other studies [6]. The statistically significant differences between the pSS and control groups were detected not only at very high frequencies but also in all other frequency ranges (low, middle, high), unlike other studies [7]. The auditory damage resulting from pSS was particularly seen at very high frequencies in the current study, which is supported by some other studies about high-frequency audiometry thresholds of pSS [6,8]. These findings may be based on indefinite pathophysiological impacts that pSS may have on the auditory system, such as effects of dryness on neurosensory elements in the inner ear. High-frequency hearing loss can be difficult to recognize in daily life, so many subjects with pSS are unaware of it. For this reason, high-frequency audiometry should be carried out routinely. It is very important for early detection of hearing loss of not only patients with pSS but also those with other autoimmune diseases. 

The present study also investigated middle ear pressures, static compliances, and statistical analyses of these values between the groups. Little research has examined tympanometry results comprehensively [7,8]. Only evidence about the type of tympanogram was presented in these studies. Not only was a type A curve obtained from all of the subjects in current study, but also the static compliances of the pSS group were lower than those of the controls. Many studies presented their results in most rheumatoid arthritis subjects as only type A tympanometry curves [8], but the tympanic membrane structure, motility, or middle ear fluids and muscle mechanisms can suffer in cases of pSS. Similarly, some studies reported that conductive hearing loss was observed in some subjects and they supposed that it could be due to dryness of the mucous membranes of the Eustachian tube and middle ear fluids [20].

Acoustic reflex thresholds were obtained in the normal range for both groups; therefore, retrocochlear pathology was not considered for the subjects with pSS. The acoustic reflex thresholds at high frequency are higher than the low ones, which was expected as that is consistent with increased high-frequency hearing thresholds in the pSS group.

The results of DPOAEs in all frequency ranges were observed in the pSS group, but one point to be noted is that response amplitudes of the pSS group were 6 kHz. This is consistent with decreased hearing thresholds towards high frequencies in pSS. There was no statistically significant difference for amplitudes between the pSS and control groups, so OHC functions and cochlear nonlinearity mechanisms can be considered normal in the pSS group. This may be explained by minimal or mild hearing loss in the pSS group, because of the hearing loss degrees, and so the functions may not suffer. About half of our subjects had minimal to mild hearing loss and these otoacoustic emission amplitudes can possibly decrease gradually to high frequencies because of the hearing loss progressing towards very high frequencies, like in some other studies [21].

Finally, it may be the most important point of this study that we investigated the auditory efferent neural pathway of pSS patients since there is a huge need for such evaluations in autoimmune diseases. Because only DPOAEs indicate that there are no direct implications for normal functions of the MOC efferent system, the present study explored the suppression of emissions to investigate the medial olivocochlear system in pSS should an abnormality of this system be suspected. Disorders of the MOC efferent system, which can be evaluated by suppression of OAEs, cause abnormalities of some auditory neural functions. Nevertheless, in this study, the demonstration of the normal functioning of the MOC reflex in subjects with pSS may indicate that the olivocochlear efferent system is functional.

Some other physiological changes such as atrophy of the spiral ganglion cells due to pSS cause sensorineural hearing loss; therefore, subjects with pSS may have neurological symptoms. The several autoantibodies that regulate the immune system suffer from autoimmune diseases; therefore, multiple problems like polyneuropathy may be revealed [22,23]. 

Ototoxicity is reported as a rare side effect of antimalarial drugs [24]. To clarify whether HCQ exposure interfered with the study results we performed subgroup analyses. Based on our subgroup analyses, exposure to HCQ did not seem to significantly affect the auditory test results of pSS patients in this study. 

In conclusion, these findings suggest that there is no synchronization problem in the efferent system, but the knowledge about the central afferent auditory system can be assumed only by acoustic reflexes in the present study. On the other hand, there is a need for more audiological evaluations, including wide-scale auditory brainstem response assessment, to see whether there is a synchronization problem in the afferent auditory system in subjects with pSS. Consequently, medical experts should refer subjects with pSS for extensive hearing assessments periodically for detecting possible audiological damage. Further studies should comprehensively investigate the auditory neural pathways not only in pSS but also in all other autoimmune diseases.
